# Recovery of Smell and Taste in Patients With Persistent COVID-19-Related Hyposmia and Dysgeusia by Targeting Inflammation and Endothelial Dysfunction

**DOI:** 10.7759/cureus.54925

**Published:** 2024-02-26

**Authors:** Maurizio G Vento, Caterina Marinelli, Luciano Ferrari, Giuseppe Pedrazzi

**Affiliations:** 1 Department of Otolaryngology, Fidenza Hospital, Parma, ITA; 2 Department of Medicine and Surgery, University of Parma, Parma, ITA

**Keywords:** sars-cov-2, post-covid-19 condition, coronavirus, covid-19, anosmia, ageusia

## Abstract

Purpose

New-onset loss of olfaction and/or taste is now recognized among the hallmark symptoms of COVID-19. In most patients, these symptoms resolve completely and spontaneously within days. However, some patients experience persistent olfactory and gustatory dysfunction after COVID-19 resolution. We evaluated the efficacy of a treatment combining several therapeutic agents to target inflammation and endothelial dysfunction in patients with persistent hyposmia and dysgeusia.

Methods

This 12-month observational pilot study involved patients presenting with symptoms of hyposmia and dysgeusia 30 days after COVID-19 had subsided. The main objective was to evaluate the efficacy of a combination of systemic corticosteroids, a glycosaminoglycan (GAG)-based antithrombotic (mesoglycan), a diuretic, and a vitamin complex. The perceived extent of olfaction and taste impairment was assessed using an 11-point visual analog scale (VAS), where 0 = complete loss of olfaction/taste and 10 = complete recovery of olfaction/taste.

Results

Eighty-seven patients with post-COVID-19 hyposmia and dysgeusia were enrolled. At treatment start (T0), the mean VAS scores were 2.0 and 3.2 for olfactory and gustatory functions, respectively. Both functions appeared to improve progressively and significantly from T0 to 12 months. A shorter time between viral infection and the start of treatment was associated with a more pronounced recovery of both senses.

Conclusions

Combined systemic corticosteroid, GAG-based antithrombotic agent (mesoglycan), and diuretic may constitute an option for treating persistent hyposmia and dysgeusia associated with COVID-19. To ensure optimal recovery, early treatment start is recommended. The described treatment protocol deserves to be further evaluated.

## Introduction

Since the early days of the coronavirus disease 19 (COVID-19) pandemic outbreak, several reports worldwide have described the occurrence of olfactory and gustatory dysfunction in infected patients [1,2]. These sensory symptoms were usually overlapping and appeared to occur early, before the onset of the respiratory syndrome, leading to the hypothesis that they may represent early diagnostic markers of severe acute respiratory syndrome coronavirus 2 (SARS-CoV-2) infection [2-5]. As a consequence, new-onset loss of olfaction (anosmia) and taste (ageusia) has been included by international scientific societies among the key symptoms of COVID-19.

Though highly variable among countries, published data on the prevalence of olfactory and gustatory dysfunction have shown a substantial prevalence of these disturbances among patients infected with SARS-CoV-2, ranging from 20% to 90% [1,3,6-10]. A meta-analysis of 24 studies involving 8,438 patients with COVID-19 from 13 countries found that the pooled rates of patients presenting with olfactory and gustatory dysfunction were 41.0% and 38.2%, respectively [11]. Another meta-analysis addressing the association between COVID-19 and self-reported loss of smell found a 62% prevalence of olfactory dysfunction in patients with COVID-19 [12]. It is currently unclear whether COVID-19-related smell and taste impairments are transient or permanent. The available evidence suggests that these symptoms completely and spontaneously resolve in most patients within approximately two weeks [1,4,7,8,13]. However, partial resolution or persistence of unchanged anosmia has been reported in a relevant proportion of patients following recovery from COVID-19 [4,13,14]. The impact of long-term smell and taste disturbances on daily-life behaviors, morbidity, and quality of life can be considerable [15-19].

The association between smell dysfunction and viral infections, including coronavirus infections, has long been known [8,20,21]. However, SARS-CoV-2 seems to differ from other respiratory viruses, as COVID-19-associated anosmia often precedes the onset of respiratory symptoms and is not associated with nasal congestion and rhinorrhea [8,20]. The exact pathogenesis of smell and taste impairments in COVID-19 is not fully elucidated. Current views concerning smell loss suggest that SARS-CoV-2 has both a direct effect on olfactory sensory neurons and an indirect effect arising from the infection of supporting cells and pericytes of the olfactory epithelium and its subsequent inflammation [20-23]. Similarly, the loss of gustatory function may arise from the indirect damage of taste receptors via the infection of epithelial cells in the mucosa of the oral cavity [20]. Neurosensory manifestations of COVID-19, such as loss of olfaction, taste, or both, may also be caused by the altered function of endothelial cells [24]. Indeed, according to a recent hypothesis about the mechanisms underlying COVID-19, viral infection activates endothelial cells to defensive mechanisms that may result in endotheliitis and contribute to microthrombosis and local tissue damage [25,26].

The endothelial glycocalyx, composed of highly-polar glycosaminoglycan (GAG) chains, proteoglycans, and glycoproteins, supports numerous physiological and pathological vascular processes [27]. The integrity of the glycocalyx is essential for normal endothelial barrier function, coagulation, complement activation, and vascular homeostasis [28,29]. Exogenous GAGs aid glycocalyx recovery, endothelial restoration, and modulation of the inflammatory response and the coagulation system following disruption of the glycocalyx through injury, together with anti-apoptotic and anti-senescence effects on endothelial cells [28-31]. Notably, infections from invading pathogens, including COVID-19 28, also promote glycocalyx disruption from endothelial cells.

Mesoglycan is a natural GAG preparation composed primarily of heparan sulfate and dermatan sulfate. Mensah et al. demonstrated in 2017 the role of exogenous heparan sulfate in glycocalyx regeneration and vasculoprotective endothelial restoration in a cell culture model of degraded glycocalyx [28]. In addition to a potential role in regenerating the glycocalyx and restoring endothelial function after COVID-19 infection, mesoglycan has antithrombotic, antioxidant, profibrinolytic, wound healing, anti-inflammatory, and antiedema properties [28,30-34].

The treatment of persistent postviral smell and taste loss is challenging, and published literature about possible therapeutic strategies is limited. A two-phased treatment protocol combining several therapeutic agents (systemic corticosteroids, a GAG-based antithrombotic (mesoglycan), a diuretic, and a vitamin complex) that target inflammation, thrombosis, and edema have been used in clinical settings to treat sudden hearing loss.

This article reports the results of an observational study evaluating the efficacy of this strategy in patients who all presented with persistent smell and taste impairments (hyposmia and dysgeusia) after COVID-19 had subsided. The rationale of the combined therapy was based on current knowledge of the effects of SARS-CoV-2 infection on tissues, namely, altered inflammatory response and vascular/endothelial and coagulation abnormalities.

## Materials and methods

Study design and patients

This observational study involved patients who reported symptoms of hyposmia and dysgeusia 30 days after the resolution of COVID-19. The main objective of this pilot study was to evaluate the efficacy of a protocol of supportive care based on the combination of a corticosteroid, GAG-based antithrombotic agent (mesoglycan), diuretic, and vitamin complex. A double-blind, controlled study design was not considered ethically appropriate as no pharmacological therapy was available for comparison and ethical approval would not have been granted for a control group without therapy. Data were collected retrospectively from patients presenting at the otolaryngology clinic of the Fidenza Hospital (Palma, Italy) in the period 2020-2021 who met the following criteria: age ≥ 18 years; clinical diagnosis of post-COVID-19 hyposmia and dysgeusia; and prior and resolved SARS-CoV-2 infection. Exclusion criteria were age < 18 years, current use of anticoagulants, and presence of clinically relevant conditions. At the first visit, the patient’s medical history was collected. In addition, patients underwent nasal endoscopy to inspect the mucosa and to determine whether rhinitis, polyposis, nasal septum deviation, or turbinate hypertrophy were present. As these conditions may also result in olfactory loss, their presence had to be excluded to avoid interference with the outcomes of the evaluated treatment. Before starting treatment, blood tests were performed, including COVID-19 serology, molecular COVID-19 testing, and testing for other viral infections (cytomegalovirus, toxoplasmosis, mononucleosis). Virological tests were required to be negative in all patients included in the study.

The study was approved by the ethics committee of the study center (Ospedale Fidenza, AUSL Parma, Italy; Comitato Etico Area Vasta Emilia Romagna). Prior to treatment start, all enrolled patients signed an informed consent. The study was conducted in accordance with the principles of the Declaration of Helsinki (2013 revision) and with national and regional standards of care.

Treatment protocol

The treatment protocol was designed based on the strategy used at the study center to manage sudden sensorineural hearing loss (hypoacusia) in accordance with current guidelines for managing this condition [24,35]. The treatment protocol designed for hyposmia and dysgeusia consisted of two phases: a seven-day first phase, during which treatment was administered in a day hospital setting, and a 30-day phase, during which therapy was self-administered at home. During the first phase of the protocol, the daily regimen was as follows: corticosteroid (dexamethasone 8 mg, administered intravenously); GAG-based antithrombotic agent (mesoglycan 30 mg, intramuscularly); multivitamin complex (ascorbic acid, vitamin B1, vitamin B2, vitamin B6 in 100 mL of physiological solution, intravenously); diuretic (carbonic anhydrase inhibitor, acetazolamide 250 mg, administered orally after blood pressure control); oral multivitamin complex with elevated zinc content (10 mg); and proton pump inhibitor (omeprazole 40 mg, intravenously). During the second phase of the treatment, the daily regimen was administered orally and consisted of corticosteroid (prednisone 5 mg for three days and then 2.5 mg for four days, after eating; GAG-based antithrombotic agent (mesoglycan 50 mg capsule, once daily for 30 days; and multivitamin complex with elevated zinc content for 30 days).

Outcomes and assessments

The hypothesis underlying this study was that the evaluated treatment protocol was able to improve post-COVID-19 hyposmia and dysgeusia. As per the treatment protocol, patients were observed for 12 months. At treatment start (T0), patients underwent an otolaryngologic examination, during which the perceived extent of olfaction and taste impairment was assessed using an 11-point visual analog scale (VAS), with a VAS score = 0 indicating complete loss of olfaction/taste, and VAS score = 10, complete recovery of olfaction/taste. The VAS, which was selected as an assessment tool in this pilot study for its ease of use, was administered to patients in paper format. Patients were asked to indicate the point on the line corresponding to their perceived loss (at T0) or recovery (after treatment start) of olfaction and taste. Olfactory and gustatory functions were assessed separately using the 11-point paper VAS.

The otolaryngologic assessment and the VAS evaluations were repeated at seven days (T1), one month (T2), three months (T3), six months (T4), and 12 months (T5) from treatment start.

Statistical analysis

The required sample size of the study was estimated using G*Power (v. 3.1.9.7; The G*Power Team, Germany) and R (v. 4.1.0; R Development Core Team, Vienna, Austria) with WebPower package (v. 0.9.2). Assuming a comparison for paired data and repeated measures between the data collected at T0 versus endpoint (T5) data, a significance level α = 0.05, power = 0.80, a Cohen effect size f = 0.40 (large effect), and the adequate sample size was at least 82 patients. The Cohen effect size f = 0.40 defined the least significant effect for the study’s aims.

Sample characteristics, including age, gender, and time from infection with SARS-CoV-2 to the start of the study treatment (T0), were analyzed by descriptive statistics (frequency, mean values, standard deviations). Patients were stratified into five age classes (≤ 30 years; 31-40 years; 41-50 years; 51-60 years; > 60 years). Patients were also stratified according to the time from the COVID-19 onset to the start of hyposmia/dysgeusia treatment as follows: ≤ 90 days (subgroup A); 91-180 days (subgroup B); 181-360 days (subgroup C); and > 360 days (subgroup D). Statistical analyses included repeated measure analysis of variance (ANOVA), univariate ANOVA, post-hoc test, and effect size determination (Cohen d effect size, f, and eta squared). Analyses were performed using Statistical Product and Service Solutions (SPSS, version 28; IBM SPSS Statistics for Windows, Armonk, NY) and Jamovi (version 2.3.26; The Jamovi Project, Newcastle, Australia) software. Response to therapy (i.e., the mean changes in the VAS for both smell and taste recovery) from T0 to T5 was analyzed using ANOVA with repeated measures, with post-hoc analysis of the latency range and t-test for paired data. The model’s assumptions (e.g., sphericity) were also checked, and appropriate corrections (Huynh-Feldt) were applied in case of violations.

## Results

Characteristics of the study population

Overall, 87 patients with post-COVID-19 hyposmia and dysgeusia were included in this observational analysis. All patients were free of all other symptoms related to COVID-19. Overall, 60.8% of patients fell within the same range of taste and smell loss. There was a slight predominance of females (57.5%), and patients aged > 60 years were a minority (8.0%), while the other age categories were equally represented (Table 1). At T0, the mean VAS scores were 2.0 and 3.2 for olfactory and gustatory function, respectively, with no significant difference between subgroups A-D.

**Table 1:** Baseline demographic and clinical characteristics. SD = Standard deviation, VAS = Visual analog scale, where 0 = complete loss of olfaction/taste and 10 = complete recovery of olfaction/taste

Characteristics	n = 87
Gender, n (%)	
Female	50 (57.5)
Male	37 (42.5)
Age, mean, y	
≤ 30	19 (21.8)
31–40	21 (24.1)
41–50	21 (24.1)
51–60	19 (21.8)
> 60	7 (8.0)
Time from infection to treatment (days), n (%)	
≤ 90 (Subgroup A)	19 (21.8)
91–180 (Subgroup B)	16 (18.4)
181–360 (Subgroup C)	40 (46.0)
> 360 (Subgroup D)	12 (13.8)
VAS score at treatment start, mean ± SD	
Smell	2.21 ± 1.99
Taste	3.17 ± 2.79

Recovery of olfaction and taste

**Figure 1:**
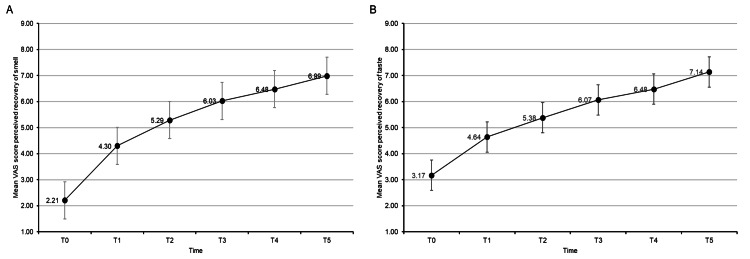
Changes in the mean visual analog scale (VAS) score from treatment start (T0) to one year. (A) Mean VAS score for perceived recovery of smell. (B) Mean VAS score for perceived recovery of taste. T0 (baseline), T1 (7 days), T2 (1 month), T3 (3 months), T4 (6 months), T5 (12 months)

There was a progressive and significant recovery of olfaction and taste. The changes in the mean VAS scores of the perceived improvement of smell and taste function over the one-year observation period are shown in Figure 1.

The differences in mean VAS scores were statistically significant (p < 0.001) between all time points in (A) and (B): T0 (baseline), T1 (7 days), T2 (1 month), T3 (3 months), T4 (6 months), and T5 (12 months).

Both functions appeared to improve progressively from T0 to T5 (Table 2), with changes in VAS scores being statistically significant (p < 0.001 for both olfaction and taste) between all the different time points.

**Table 2:** Univariate analysis of variance (ANOVA) of treatment effects on smell and taste over time. ^a^A ≤ 90 days, B 91–180 days, C 181–360 days, D > 360 days

Function	Sum of squares (SSBS; SSWS)		df		Mean of square (MSBS; MSWS)	F	P	Tukey’s post-hoc comparisons^a^ (P)
Smell								
T0 (baseline)	35.838; 306.438	3; 83		11.946; 3.692		3.236	0.026	NS
T1 (7 days)	59.219; 509.011	3; 83		19.740; 6.133		3.219	0.027	A>D (0.018)
T2 (1 month)	122.202; 506.574	3; 83		40.734; 6.103		6.674	<0.001	A>C (0.018); A>D (<0.001)
T3 (3 months)	118.804; 534.592	3; 83		39.601; 6.441		6.148	<0.001	A>C (0.013); A>D (<0.001)
T4 (6 months)	137.474; 559.250	3; 83		45.825; 6.738		6.801	<0.001	A>C (0.016); A>D (<0.001); B>D (0.029)
T5 (12 months)	112.759; 595.988	3; 83		37.586; 7.181		5.234	0.002	A>D (<0.002); B>D (0.017)
Taste								
T0 (baseline)	38.264; 630.150	3; 83		12.755; 7.592		1.680	0.178	NS
T1 (7 days)	55.221; 572.624	3; 83		18.407; 6.899		2.668	0.053	A>D (0.038)
T2 (1 month)	125.587; 607.395	3; 83		41.862; 7.318		5.720	0.001	A>B (0.018); A>C (0.018); A>D (0.002)
T3 (3 months)	119.244; 588.342	3; 83		39.748; 7.088		5.607	0.001	A>B (0.044); A>C (0.048); A>D (<0.001)
T4 (6 months)	121.077; 567.647	3; 83		40.359; 6.839		5.901	0.001	A>C (0.045); A>D (<0.001)
T5 (12 months)	120.875; 601.079	3; 83		40.292; 7.242		5.564	0.002	A>D (<0.001); B>D (0.024); C>D (0.032)

Effect of time from infection to treatment start on outcomes

The time from SARS-CoV-2 infection to treatment started affected the outcomes.

Effects of time and latency range on smell and taste

An analysis evaluating the combined effect of time spent in the study and the factor relating to the time elapsed between COVID-19 infection and admission to the clinic divided patients into four subgroups corresponding to the following values: (A: ≤ 90 days; B: 91-180 days; C: 181-360 days, and D: > 360 days). Post-hoc ANOVA using Tukey’s test revealed significant differences between the subgroups, in particular between group A (admission to the Otolaryngology/Ear, Nose, and Throat Department within three months) and group D (admission after more than 12 months). Patients treated earlier after the onset of infection when neurosensory damage was less pronounced recovered smell and taste more rapidly and to a greater extent than when intervention was taken later after the resolution of COVID-19.

**Figure 2:**
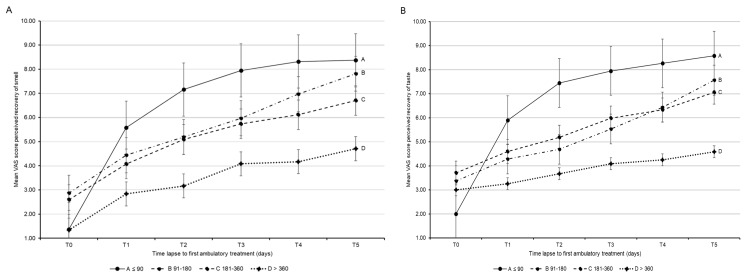
Changes in the mean visual analog scale (VAS) score from treatment start (T0) to one year after stratification of patients according to time from SARS-CoV-2 infection to treatment start. (A) Mean VAS score for perceived recovery of smell; (B) Mean VAS score for perceived recovery of taste A ≤ 90 days, B 91–180 days, C 181–360 days, D > 360 days to first ambulatory treatment: T0 (baseline), T1 (7 days), T2 (1 month), T3 (3 months), T4 (6 months), T5 (12 months)

Figure 2 shows the VAS changes in the four subgroups (A-D) considered over the one-year observation period and showed that both olfaction (Figure 2A) and taste (Figure 2B) improved more substantially the shorter the interval between viral infection and the start of treatment. In detail, at T0, the differences in mean VAS scores for recovery of both smell and taste were not statistically significant, while at T1 (seven days from treatment start), patients treated within 90 days from SARS-CoV-2 infection (group A) reported significantly greater improvements in olfaction and taste compared with patients treated after > 360 days from infection (group D).

At T2 (one month from treatment start) and T3 (three months), the patients of group A had significantly better improvement in olfaction than patients treated after > 180 days from viral infection. With regard to improvements in taste, they were significantly greater in group A than in any other group at T2 and T3. At T4 (six months), group A patients had significantly greater improvement in both olfaction and taste compared with group C and D patients, but not with group B patients; group B patients had significantly greater improvement in olfaction than group D patients.

Finally, after one year (T5), patients of groups B and C reported further improvements in olfaction and taste, getting close to the scores of group A; patients treated after > 360 days from infection (group D) showed less substantial improvements in olfaction and taste, with differences being statistically significant versus patients treated within 180 days from infection (groups A and B). Of note, in patients treated within 90 days from COVID-19 onset (group A), improvements in smell and taste appeared to plateau around three months of observation (T3), while the other groups showed an increasing trend of functional recovery over the entire observation period, without reaching the improvement achieved by group A patients (Figures 2A-2B).

Evaluation of treatment effects on smell over time

To analyze the effects at individual times (T), analyses were carried out using univariate ANOVA, placing the expressed evaluation of the VAS scale as the dependent variable and the temporal latency range for accessing the clinic with the application of the tests as a factor post hoc. The results of the analysis (Table 2) showed the following:

(a) At T0 (baseline), the average ratings between the four subgroups of patients do not present significant differences.

(b) At T1 (seven days), the patients in group A (taken care of within 90 days) already show significant differences in the improvement process compared to the patients in group D (over 360 days).

(c) At T2 (one month) and T3 (three months), the patients in group A (≤ 90 days) show an improvement, which leads them to have significantly higher mean scores on the VAS scale compared to patients treated late (> 180 days).

(d) At T4 (six months), the VAS scale assessments of patients in group A are higher than those in groups C (181-360 days) and D (> 360 days). Patients in group B (91-180 days) differ from patients in group D (> 360 days) with significantly higher scores.

(e) At T5 (endpoint: 12 months), it is observed that the scores of patients in classes B and C show a further improvement in their quality of life, coming close to the patients in group A. Patients taken into care after more than 360 days from COVID-19 infection show a minor improvement at the endpoint and differ statistically from those treated within 180 days (A and B).

Evaluation of treatment effects on taste over time

Univariate ANOVA was carried out in the same manner as that carried out for the recovery of smell, setting as the dependent variable the assessments carried out with the VAS scale carried out in the various steps of taking charge of the patient (from T0 to T5) and using as a factor the variable relating to the time latency from COVID infection to inclusion in the study. The results of the statistical analysis (Table 2) highlighted the following aspects:

(a) At T0 (baseline), there are no significant differences in the mean ratings between the four subgroups of patients.

(b) At T1 (seven days), the patients in group A (taken care of within 90 days) already show significant differences in the improvement process compared to the patients in group D (over 360 days).

(c) At T2 (one month) and T3 (three months), the patients in group A (≤ 90 days) show an improvement, which leads them to have significantly higher mean scores on the VAS scale than all the other groups.

(d) At T4 (six months), the VAS scale assessments of patients in group A are higher than those in groups C (181-360 days) and D (>360 days). However, patients in group B (91-180 days) do not show significant differences compared to those taken earlier.

(e) At T5 (endpoint: 12 months), it is observed that the scores of patients in classes B and C show a further improvement in their quality of life, coming close to the patients in group A.

Patients taken into care after more than 360 days from the date of COVID-19 infection show a minor improvement at the endpoint and differ statistically from all other groups.

Comparison of treatment effects on olfactory and gustatory function

**Figure 3:**
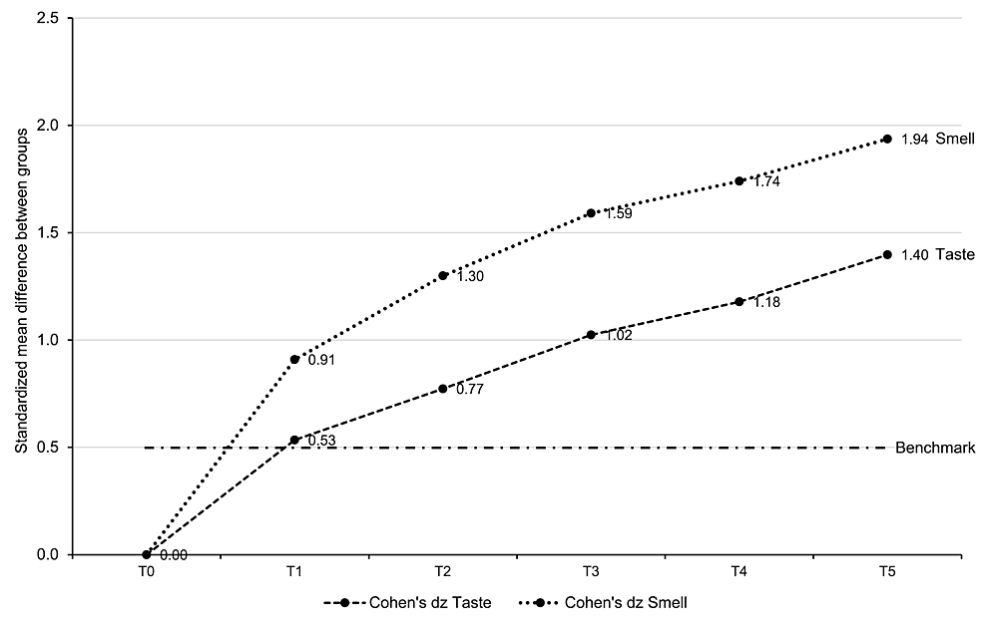
Comparison of the effects of treatment on smell and taste function over one year. Cohen’s dz statistical test for paired samples. T0 (baseline), T1 (7 days), T2 (1 month), T3 (3 months), T4 (6 months), T5 (12 months)

Treatment effects were more pronounced on olfactory function than on gustatory function. Figure 3 summarizes the results of the comparison of the improvements reported from T0 to T5 in smell and taste functions. The reported recovery of olfaction appeared more substantial than the recovery of gustatory function throughout the observation period.

Safety

No safety problems were identified, and the treatment, already well-established in the specialist field of otolaryngology for sudden hearing loss, was always well-tolerated.

## Discussion

We report the results of a pilot study investigating the efficacy of a treatment combining several agents on the recovery of smell and taste in patients with persistent olfactory and gustatory dysfunction related to COVID-19 who were followed up for 12 months. We note that almost 60% of patients in the study were women, consistent with other studies suggesting that females are more susceptible to developing olfactory and gustatory dysfunction than males [4,8,11,23,36]. This may reflect gender differences in the processes of inflammatory reactions. The treatment was associated with a continuous and significant recovery of both smell and taste from baseline to 12 months, as evidenced by the statistically significant increases in mean VAS scores from 2.0 at baseline to 6.9 at 12 months for smell and from 3.2 to 7.1 for taste.

Improvements in both sensory functions were particularly pronounced during the first three months of observation. Notably, the analysis of smell and taste recovery based on the time from SARS-CoV-2 infection to the treatment of sensorineural dysfunction showed that patients treated within 90 days from viral infection had a more rapid and substantial recovery (final VAS scores for the recovery of both functions were > 8 compared to patients receiving treatment at > 90 days after viral infection. The benefits of early intervention have also been shown in studies of mesoglycan in audiovestibular disorders of vascular origin [37]. We hypothesize that, similarly, the earlier therapy is initiated in relation to a pathological event such as SARS-CoV-2 viral damage to the acoustic nerve, the greater the chance of recovery.

Another important point highlighted by this study is that, based on the effect size analysis, the evaluated treatment was more effective on smell than on taste function. Overall, the patients treated in the present study did not achieve a complete recovery of smell and taste over the 12-month observation period. For the entire population, the mean VAS scores at 12 months ranged between 6.9 and 7.1, while the subgroup with the best response to treatment (those treated within 90 days from COVID-19 onset) achieved mean VAS scores of 8.58 for taste and 8.37 for smell. Given the increasing trend of the observed time course of VAS score changes, it is possible that longer observation times would have revealed additional improvements in smell and taste function.

We developed the treatment regimen evaluated based on a protocol used in our clinic to treat sudden sensorineural hearing loss, assuming that sensorineural conditions caused by viral infection may share common mechanisms. We also took advantage of current knowledge of the pathophysiology of SARS-CoV-2 infection, including excessive inflammatory response, edema of the infected tissues, endothelial dysfunction, and microthrombus formation, which may also play a role in the damage to the olfactory and gustatory epithelia and other relevant tissues [5,14,15,20,23-26]. Based on this rationale, the combined regimen included a potent anti-inflammatory agent (intravenous dexamethasone followed by oral prednisone), a diuretic (acetazolamide), and an antithrombotic agent (intramuscular mesoglycan, followed by oral mesoglycan).

Mesoglycan, as a natural GAG compound extracted from porcine intestinal mucosa and consisting of heparan sulfate, dermatan sulfate, an electrophoretically slow-moving type of heparin, and chondroitin sulfate [34], has several properties relevant to olfactory and gustatory dysfunction related to SARS-CoV-2. The compound, which has anti-inflammatory, antithrombotic, profibrinolytic, antioxidant, and antiedema properties, has been shown in preclinical and clinical studies to improve the microcirculation and reduce edema through restoration of endothelial function [28,30-33,38]. In addition, mesoglycan has anti-apoptotic and anti-senescence effects on endothelial cells [29,31].

The well-documented association between COVID-19 and impaired olfaction and taste and the recognition of these manifestations as distinctive symptoms of SARS-CoV-2 infection has undoubtedly contributed to the recently increased awareness of changes affecting the senses of smell and taste. These alterations usually receive less attention in clinical practice than vision or hearing impairments, and their impact on patient quality of life is often underestimated or overlooked [15]. However, evidence from several studies suggests that the effects of olfactory and gustatory dysfunctions are relevant and wide-ranging [19]. For example, a systematic literature review found a reciprocal association between depression and olfactory dysfunction and highlighted that depressive symptoms worsen with the severity of olfaction impairment [39]. A United Kingdom survey among people with olfactory disorders found elevated rates of depression and anxiety (43% and 45%, respectively), problems with eating (92%), difficulties in relationships (54%), and social isolation (57%) [18].

The poor recognition of the impact of smell and taste disorders has also contributed to the lack of studies assessing potential therapies and evidence-based guidelines for management. In recent years, probably due to the increased prevalence of these disorders related to the COVID-19 pandemic, several efforts towards increasing awareness and improving management have been made (especially for olfactory dysfunction). The current consensus among clinical olfactory experts is that olfactory training has an established position in the management of smell loss and is overall beneficial, while evidence supporting the use of medical treatment, including systemic and topic corticosteroids, is weak [14,15,36,40]. There is strong agreement on the importance of early treatment (olfactory training, as well as corticosteroid treatment) at the onset of dysfunction, when neurosensory changes may still be reversible [41-43]. Overall, the need for studies evaluating corticosteroids and other medications for treating smell loss is widely acknowledged.

The published evidence on the management of post-COVID-19 olfactory and gustatory loss is still limited to small studies and case series [44-47]. According to the latest version of a living systematic Cochrane review of interventions for the treatment of persistent post-COVID-19 olfactory dysfunction, the study results currently available do not allow meaningful conclusions to be drawn [48]. However, their review identified several ongoing trials, the results of which will soon be available. An international expert group in clinical olfaction has called for caution in the use of systemic corticosteroids in early COVID-19-related olfactory dysfunction, pointing out that the evidence of their usefulness is weak, the rate of spontaneous recovery of olfaction is high, and corticosteroids are associated with several adverse events [14]. This group has recommended smell training, which should, however, be implemented at the onset of olfactory dysfunction [14]. An expert panel composed of members of the British Rhinological Society and ENTUK has recently issued consensus statements according to which olfactory training is recommended for all patients with persistent loss of smell of greater than two weeks duration, while oral steroids, steroid rinses, and omega 3 supplements may be considered in specific cases [49].

Our study has several limitations inherent to its observational and exploratory nature, including the lack of a control arm. Therefore, it cannot be excluded that the observed improvements in smell and taste reflect the natural course of recovery. In this respect, available evidence suggests that time is an important factor and that repair and recovery of a functional olfactory epithelium following SARS-CoV-2 infection might be a time-demanding process [4]. The fact that smell and taste recovery were assessed only subjectively is another limitation of the present study. Evidence has shown that objective and subjective measures of olfactory function are often discrepant and that patients tend to underestimate smell recovery [50].

## Conclusions

The results of this pilot study suggest that combined systemic treatment with corticosteroids, GAG-based antithrombotic (mesoglycan), and diuretic agents may constitute an option for the treatment of persistent hyposmia and dysgeusia associated with SARS-CoV-2 infection. To ensure optimal recovery of the senses of smell and taste, treating these neurosensory dysfunctions should not be postponed. The strategy proposed here deserves further evaluation in an adequately designed study. Overall, further research is needed to identify efficacious strategies for treating COVID-19-related neurosensory symptoms because of their potentially high prevalence and impact on patient quality of life.
